# Effect of Myricetin, Pyrogallol, and Phloroglucinol on Yeast Resistance to Oxidative Stress

**DOI:** 10.1155/2015/782504

**Published:** 2015-04-27

**Authors:** Vanda Mendes, Rita Vilaça, Victor de Freitas, Pedro Moradas Ferreira, Nuno Mateus, Vítor Costa

**Affiliations:** ^1^Instituto de Investigação e Inovação em Saúde, Universidade do Porto, Rua Alfredo Allen s/n, 4200-135 Porto, Portugal; ^2^IBMC, Instituto de Biologia Molecular e Celular, Universidade do Porto, Rua do Campo Alegre 823, 4150-180 Porto, Portugal; ^3^ICBAS, Instituto de Ciências Biomédicas Abel Salazar, Departamento de Biologia Molecular, Universidade do Porto, Rua de Jorge Viterbo Ferreira 228, 4050-313 Porto, Portugal; ^4^REQUIMTE/LAQV, Faculdade de Ciências da Universidade do Porto, Rua do Campo Alegre s/n, 4169-007 Porto, Portugal

## Abstract

The health beneficial effects of dietary polyphenols have been attributed to their intrinsic antioxidant activity, which depends on the structure of the compound and number of hydroxyl groups. In this study, the protective effects of pyrogallol, phloroglucinol, and myricetin on the yeast *Saccharomyces cerevisiae* were investigated. Pyrogallol and myricetin, which have a pyrogallol structure in the B ring, increased H_2_O_2_ resistance associated with a reduction in intracellular oxidation and protein carbonylation, whereas phloroglucinol did not exert protective effects. The acquisition of oxidative stress resistance in cells pretreated with pyrogallol and myricetin was not associated with an induction of endogenous antioxidant defences as assessed by the analysis of superoxide dismutase and catalase activities. However, myricetin, which provided greater stress resistance, prevented H_2_O_2_-induced glutathione oxidation. Moreover, myricetin increased the chronological lifespan of yeast lacking the mitochondrial superoxide dismutase (Sod2p), which exhibited a premature aging phenotype and oxidative stress sensitivity. These findings show that the presence of hydroxyl groups in the ortho position of the B ring in pyrogallol and myricetin contributes to the antioxidant protection afforded by these compounds. In addition, myricetin may alleviate aging-induced oxidative stress, particularly when redox homeostasis is compromised due to downregulation of endogenous defences present in mitochondria.

## 1. Introduction

Oxidative stress is a hallmark of human disorders such as cancer and age-associated diseases [1]. It results from an unbalance between the levels of reactive oxygen species (ROS) or reactive nitrogen species (RNS) and cellular antioxidant defenses. The toxicity of high levels of ROS and RNS is associated with the accumulation of damaged molecules, including proteins, lipids, and nucleic acids [1]. Under normal physiological conditions, ROS are kept at low levels by antioxidant defenses such as superoxide dismutases (SOD) that catalyze the dismutation of superoxide radicals into hydrogen peroxide, catalases, or peroxidases that reduce H_2_O_2_ into water, as well as nonenzymatic defenses, including glutathione that plays critical roles in redox homeostasis and cellular detoxification [2]. In addition, antioxidants obtained in the diet, such as vitamins C and E and phenolic compounds, play essential role in cellular protection [3].

Phenolic compounds are natural antioxidants present in the human diet through the consumption of fruits, vegetables, and drinks such as juice, tea, coffee, and wine [4, 5]. Structurally, these compounds are characterized by having one or more hydroxyl groups attached to at least one aromatic ring [4]. The number and position of hydroxyl groups are important features that affect the antioxidant activity of phenolic compounds [6]. These compounds possess antiproliferative, proapoptotic, and anti-inflammatory properties and they have been associated with the prevention of cancer and cardiovascular, neurodegenerative, and metabolic disorders [7, 8]. The protective effects of these compounds have been attributed not only to their intrinsic antioxidant activity but also to the modulation of cell signaling pathways, including mitogen-activated protein kinase cascades, which regulate oxidative stress responses [9–11].

The budding yeast* Saccharomyces cerevisiae* has been used as a eukaryotic model organism to characterize the molecular mechanisms underlying oxidative stress resistance and to evaluate the antioxidant potential of dietary extracts and phenolic compounds [12]. We have previously reported that quercetin, the most common flavonol in the diet, increases yeast oxidative stress resistance [13] and exerts its protective effects against oxidative stress by inducing the biosynthesis of trehalose, a stress protectant disaccharide, and the activation of the cell wall integrity pathway [14]. Other studies have shown that resveratrol and catechin increase oxidative stress resistance in yeast by mechanisms associated with the activation of catalase [15], whereas delphinidin 3-glucoside and petunidin 3-glucoside protect yeast through activation of the stress response regulators Msn2p and Msn4p [16]. Moreover, the sirtuin Hst3p has been implicated in oxidative stress protection afforded by a polyphenol-enriched cocoa powder [17].

**Figure 1:**
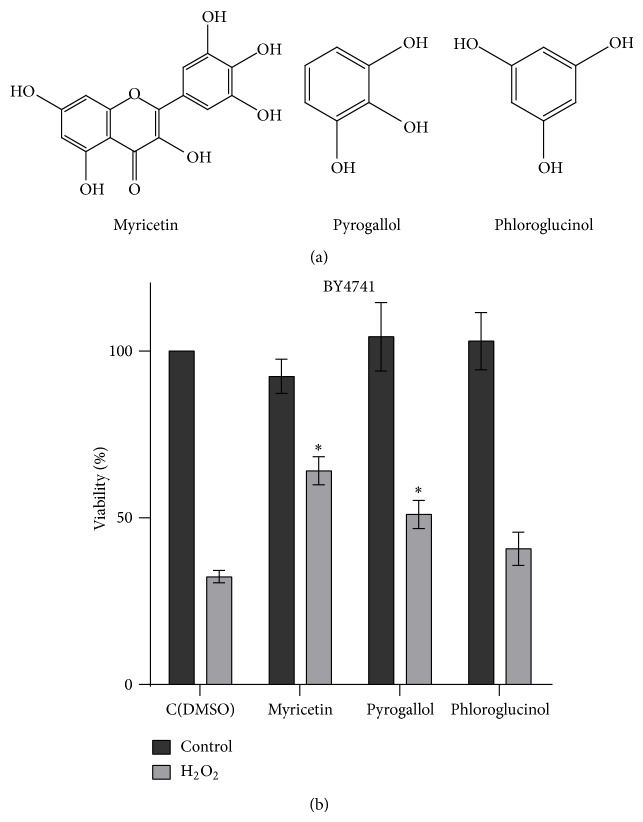
(a) Chemical structure of the polyphenolic compounds used in this work. (b) Effect of myricetin and simple phenols (pyrogallol and phloroglucinol) on oxidative stress resistance. Yeast cells were grown to the exponential phase in YPD medium, pretreated with compounds (300 *μ*M) or equal volume of DMSO (control) for 15 min, and subsequently treated with 1.5 mM H_2_O_2_ for 1 h. Viability is expressed as the percentage of the CFU. Values are mean ± SEM of at least 3 independent assays. Values were compared by one-way ANOVA, Dunnett's multiple comparisons test (^∗^ *p* < 0.05).

Pyrogallol and phloroglucinol are simple phenols that contain three hydroxyl groups in the ortho- and metaposition, respectively, of a benzene ring (Figure 1(a)). Humans are exposed to pyrogallol through ingestion of tea and coffee [18] but also from degradation of gallic acid in colon [19]. Phloroglucinol is found as a monomer of phlorotannins in brown algae, which is increasing in the human diet [20]. Myricetin is a naturally occurring flavonol characterized by having a pyrogallol structure in the B ring as well as a 4-oxo function with an unsaturated bond between the 2 and 3 carbons within the C ring and the presence of hydroxyl groups at C3 and C5 [6] (Figure 1(a)). In the human diet, myricetin is commonly found in tea, berries, and red wine [21]. In this study, we investigated the effect of myricetin, pyrogallol, and phloroglucinol on yeast resistance to oxidative stress.

## 2. Materials and Methods

### 2.1. Reagents

All reagents and chemicals used were of analytical grade. Sodium or potassium phosphates, riboflavin, and H_2_O_2_ were purchased from Merck (Darmstadt, Germany). Dimethyl sulfoxide (DMSO), myricetin, pyrogallol, phloroglucinol, and nitroblue tetrazolium were purchased from Sigma (Sintra, Portugal). Phenolic compounds were dissolved in DMSO at a 200 mM stock concentration and stored at −80°C. Solutions were prepared in ultrapure water (Milli-Q).

### 2.2. Yeast Strains and Growth Conditions

*Saccharomyces cerevisiae* cells (Euroscarf, Germany) used in this study were BY4741 (Mat*α*,* his3*Δ_1_,* leu2*Δ_0_,* met15*Δ_0_,* ura3*Δ_0_; parental strain),* sod1*Δ (BY4741* sod1*Δ::*KanMX4*), and* sod2*Δ (BY4741* sod2*Δ::*KanMX4*). Yeast cells were grown in YPD medium [1% (w/v) yeast extract, 2% (w/v) bactopeptone, and 2% (w/v) glucose] or in synthetic complete (SC) drop-out medium containing 2% (w/v) glucose, 0.67% (w/v) yeast nitrogen base without amino acids supplemented with the appropriate amino acids (80 mg His L^−1^, 400 mg Leu L^−1^, and 80 mg trp L^−1^), and nucleotides (80 mg Ura L^−1^). Cultures were maintained in an orbital shaker, at 26°C and 120 rpm, with a ratio of flask volume/medium volume of 5 : 1.

### 2.3. Oxidative Stress Resistance Assays

Yeast cells were grown to the exponential phase (OD_600_ = 0.5-0.6) in YPD medium, pretreated with polyphenols (myricetin, pyrogallol, or phloroglucinol at 300 *μ*M) or equal volume of DMSO (vehicle) for 15 min, and subsequently exposed to 1.5 mM H_2_O_2_ for 1 hour. Cell viability was determined by dilution plate counts on YPD medium containing 1.5% agar. Colonies were counted after growth at 26°C for 3 days. Viability was expressed as the percentage of colony-forming units (CFU).

### 2.4. Intracellular Oxidation

The oxidant-sensitive probe 2′,7′-dichlorodihydrofluorescein (H_2_DCF-DA) (Molecular Probes) was used to measure intracellular oxidation. Yeast cells grown to the exponential phase in YPD medium and pretreated with polyphenols for 15 min were subsequently exposed to 1.5 mM H_2_O_2_ for 1 hour in the absence or presence of 25 *μ*M H_2_DCF-DA. Cells were spun down (4,000 rpm, 4 min), washed twice, and suspended in filtered phosphate-buffered saline (PBS; 137 mM NaCl, 2.7 mM KCl, 10 mM Na_2_HPO_4_, and 1.8 mM KH_2_PO_4_, pH 7.4). Fluorescence was measured in FL-1 channel (excitation and emission wavelength at 488 nm and 525 nm, resp.) in a Becton-Dickinson FACSort flow cytometer. Autofluorescence was analyzed in cells untreated with H_2_DCF-DA. Data was acquired from a total of 10,000 events/samples. BD CellQuest Pro Software was used for data acquisition and FlowJo Software for data analysis.

### 2.5. Protein Carbonylation

Protein extracts were prepared in 50 mM potassium phosphate buffer (pH 7.0) containing protease inhibitors (Complete, Mini, EDTA-free Protease Cocktail Inhibitor Tablets; Roche Applied Science), by vigorous shaking of the cell suspension, in the presence of glass beads, for 5 min. Short pulses of 1 min were used, with 1 min intervals on ice. Protein content was estimated by the Lowry method, with bovine serum albumin as a standard. Protein carbonylation assays were performed by slot-blot analysis, as previously described [13], using rabbit IgG anti-dinitrophenyl (DNP) (Sigma) at a 1 : 5,000 dilution as the primary antibody and goat anti-rabbit IgG-peroxidase (Sigma) at 1 : 5,000 as the secondary antibody. Immunodetection was performed by chemiluminescence, with a kit from GE Healthcare (RPN 2109). Quantification of bands was performed by densitometry.

### 2.6. Glutathione Levels and Enzymatic Activities

All the procedures were carried out at 4°C. Yeast cells were harvested by centrifugation. Glutathione levels were measured by the method of Tietze [22], as described in a previous work [13]. For enzyme activities, yeast extracts were prepared as described for the analysis of protein carbonylation. The activity of catalase and SOD was analyzed* in situ*, after separation of proteins (50 *μ*g) by native polyacrylamide gel electrophoresis (PAGE), as described previously [23, 24]. Quantification of bands was performed by densitometry.

### 2.7. Chronological Lifespan

Overnight cultures in SC medium were diluted to OD_600_ = 0.5 and grown to the stationary phase for 3 days (in the case of BY4741 and* sod1*Δ cells) or for 1 day (in the case of* sod2*Δ cells). Then, the compounds (300 *μ*M myricetin or pyrogallol) or DMSO (vehicle; volume identical to compounds) were added to the cultures (day 0). These cells were kept in culture media at 26°C and viability was analyzed at indicated times by standard dilution plate counts on YPD medium containing 1.5% agar. Colonies were counted after growth at 26°C for 3 days and viability was expressed as the percentage in CFU relative to day 0.

### 2.8. Statistical Analysis

Analysis was performed in GraphPad Prism. Data are expressed as the mean values ± standard error of the mean (SEM) of at least three independent experiments. The 0.05 probability level was selected as the point of statistical significance. Values of oxidative stress resistance assays were analyzed by one-way ANOVA and compared by Dunnett's multiple comparisons test. Intracellular ROS and protein carbonyls were analyzed by two-way ANOVA and compared by Sidak's multiple comparisons test. Statistical analysis of total and oxidized glutathione levels and the ratio GSSG/GSH_T_ was performed by two-way ANOVA, Sidak's multiple comparisons test (^∗^ *p* < 0.05) for comparison of values between treatments in each condition (control or H_2_O_2_), and multiple *t*-tests using the Holm-Sidak method for corrections (^∗^ *p* < 0.05) for comparison of values between control and H_2_O_2_ for all treatments. Lifespans were compared by Student's *t*-test.

## 3. Results

### 3.1. Myricetin and Pyrogallol Increase Hydrogen Peroxide Resistance in* Saccharomyces cerevisiae*

**Figure 2:**
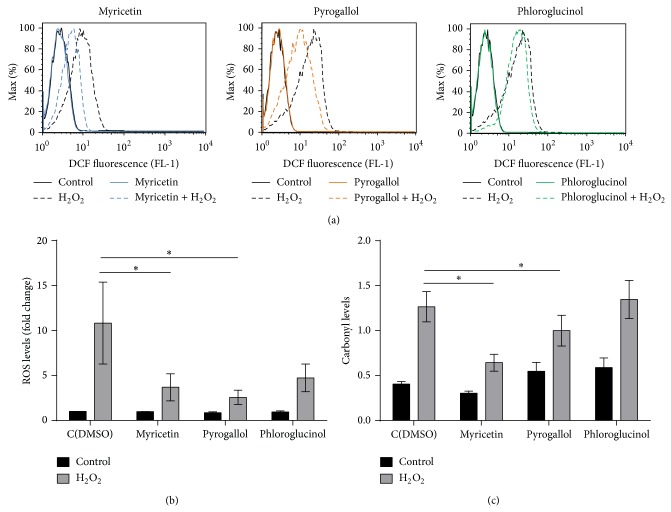
Effect of myricetin, pyrogallol, and phloroglucinol on intracellular oxidation and oxidative damage. Yeast cells were grown in YPD medium to the exponential phase and pretreated with compounds (300 *μ*M) or equal volume of DMSO (control) for 15 min and subsequently treated with 1.5 mM H_2_O_2_ for 1 h. (a) Representative histograms of intracellular ROS analyzed by flow cytometry using H_2_DCF-DA as a probe. (b) Quantification of intracellular ROS expressed by mean fluorescence intensity in 10,000 cells (arbitrary units) from at least 3 independent assays. (c) Quantitative analysis of protein carbonyl content was performed by densitometry using data taken from the same membrane. Proteins were derivatized with DNPH and slot-blotted into a PVDF membrane. Immunodetection was performed using an anti-DNP antibody. Values are mean ± SEM of at least 3 independent assays. Values were compared by two-way ANOVA, Sidak's multiple comparisons test (^∗^ *p* < 0.05).

To assess the effect of myricetin, pyrogallol, and phloroglucinol on oxidative stress resistance, exponential phase yeast cells were pretreated with these compounds individually (300 *μ*M) or DMSO (control) for 15 min and subsequently exposed to 1.5 mM H_2_O_2_ for 1 hour. The presence of polyphenols per se (in the absence of H_2_O_2_) did not affect cell viability, intracellular oxidation, or protein oxidation. Myricetin and pyrogallol, in contrast with phloroglucinol, increased cell viability from 33% (in control cells) to 64% and 51%, respectively (Figure 1(b)). To investigate if H_2_O_2_ resistance induced by these polyphenols was correlated with a decrease in oxidative stress markers, intracellular ROS levels were measured by flow cytometry using cells labeled with an oxidant-sensitive probe, H_2_DCF-DA (Figures 2(a)-2(b)), and protein oxidation was assessed through the analysis of protein carbonyl content (Figure 2(c)). In control cells, exposure to H_2_O_2_ caused a 10-fold increase in intracellular ROS and a 3-fold increase in protein carbonylation. Myricetin and pyrogallol, but not phloroglucinol, significantly decreased H_2_O_2_-induced intracellular oxidation and protein carbonylation.

### 3.2. Myricetin and Pyrogallol Do Not Affect the Activity of Superoxide Dismutase or Catalase

**Figure 3:**
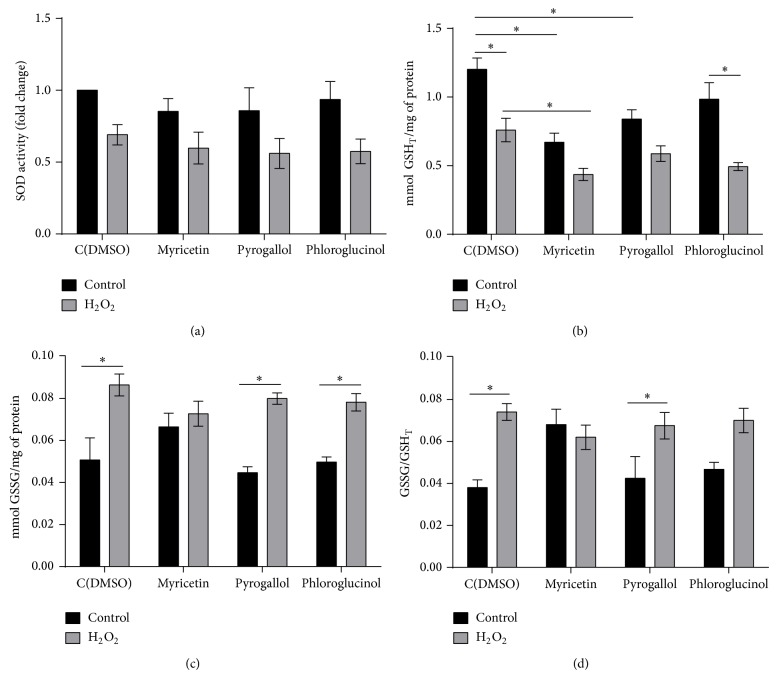
Effect of myricetin, pyrogallol, and phloroglucinol on antioxidant defenses. Yeast cells were grown in YPD medium to the exponential phase and pretreated with compounds (300 *μ*M) or equal volume of DMSO (control) for 15 min and subsequently treated with 1.5 mM H_2_O_2_ for 1 h. (a) SOD activity was assessed* in situ* after native PAGE. Band intensities were measured by densitometry using data taken from the same gel; (b) GSH_T_ levels, (c) GSSG levels, and (d) ratio between oxidized glutathione and total glutathione levels. Values are mean ± SEM of at least 3 independent assays. GSH_T_ and GSSG levels were compared by two-way ANOVA, Sidak's multiple comparisons test (^∗^ *p* < 0.05) and the ratio was compared by Student's *t*-test (^∗^ *p* < 0.05).

To investigate if the protective effect of myricetin or pyrogallol was associated with an induction of antioxidant defenses, the activity of superoxide dismutase and catalase was determined. Consistent with published data [25], SOD activity decreased 31% in control cells (DMSO-treated) exposed to H_2_O_2_ (Figure 3(a)). Pretreatment with the phenolic compounds did not affect basal SOD activity or prevent its decrease upon exposure to H_2_O_2_. Catalase is not expressed in exponential phase cells [26] and, therefore, its activity was not detected in control cells. Moreover, it was not induced in cells treated with the tested compounds (data not shown). These results indicate that the increase of oxidative stress resistance in cells pretreated with myricetin or pyrogallol did not result from the induction of SOD and catalase.

### 3.3. Myricetin Suppresses H_2_O_2_-Induced Glutathione Oxidation

The tripeptide glutathione (GSH) is the most abundant low-molecular weight thiol that serves to maintain a reduced intracellular environment [27]. To assess the effect of myricetin, pyrogallol, and phloroglucinol on redox homeostasis, glutathione levels were determined in cells exposed to H_2_O_2_ (Figures 3(b)–3(d)). In control cells, after exposure to H_2_O_2_, total glutathione levels (GSH_T_) decreased 37% whereas GSSG levels increased 70%, increasing the ratio between GSSG and GSH_T_. Similar results were observed in cells pretreated with phloroglucinol, which is consistent with the fact that this compound did not affect oxidative stress resistance. Myricetin and pyrogallol per se (in the absence of H_2_O_2_) decreased GSH_T_ levels. However H_2_O_2_-induced glutathione depletion was lower in cells pretreated with these compounds, comparing with DMSO-treated cells. Moreover, the increase in the levels of GSSG and in the ratio GSSG/GSH_T_ induced by H_2_O_2_ was suppressed by myricetin, but not by pyrogallol. This is consistent with our data showing that oxidative stress resistance in cells pretreated with myricetin was higher than the observed in pyrogallol pretreated cells.

### 3.4. Myricetin Increases the Chronological Lifespan of* sod2*Δ Mutant Cells

**Figure 4:**
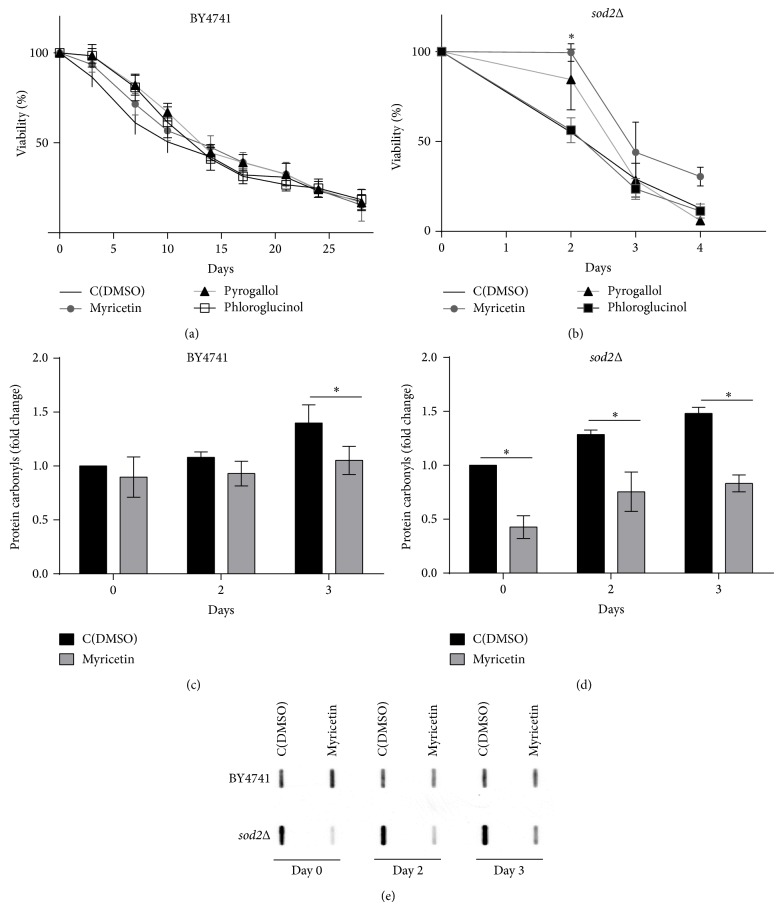
Effect of myricetin, pyrogallol, and phloroglucinol on (a) BY4741 and (b)* sod2*Δ cells CLS. Cells were grown in SC-glucose medium to the stationary phase and treated with myricetin, pyrogallol, or phloroglucinol (300 *μ*M). Viability was measured by standard dilution plate counts which were considered 100% on day 0 (first treatment day). (c, d) On the indicated days, the levels of protein carbonyls were analyzed during aging of BY4741 (c) and* sod2*Δ (d) cells pretreated with myricetin. Quantitative analysis of protein carbonyl content was performed by densitometry using data taken from the same membrane. Proteins were derivatized with DNPH and slot-blotted into a PVDF membrane. Immunodetection was performed using an anti-DNP antibody. A representative blot is shown in (e). Values are mean ± SEM of at least 3 independent assays. Viability values were compared by Student's *t*-test (^∗^ *p* < 0.05) and protein carbonyl values were compared by two-way ANOVA, Sidak's multiple comparisons test (^∗^ *p* < 0.05).

Aging has been associated with an increase in intracellular oxidation and accumulation of oxidative damage [28]. Mitochondria are a major source of ROS and its dysfunction has been implicated in aging [29, 30]. Mitochondria contain several antioxidant enzymes, including the superoxide dismutases Sod1p (CuZnSOD) that is present in the mitochondrial intermembrane space (and cytosol) and Sod2p (MnSOD) located in the mitochondrial matrix. Cells lacking Sod1p or Sod2p exhibit a decreased chronological lifespan associated with the accumulation of oxidative damage [31] (Figures 4(a)-4(b)). The protective effect of myricetin and pyrogallol against oxidative stress caused by H_2_O_2_ led us to assess its effect on the chronological lifespan (CLS) of parental cells and of* sod1*Δ and* sod2*Δ mutant cells. Parental cells showed a time-dependent loss of cell viability, which was not affected by pretreatment with myricetin, pyrogallol, or phloroglucinol (Figure 4(a)). These phenolic compounds also did not affect the lifespan of* sod1*Δ cells (data not shown). However, myricetin significantly increased the CLS of* sod2*Δ cells (Figure 4(b)), suggesting that this compound exerts a protective effect that is particularly relevant in cells that have a decreased capacity to scavenge superoxide radicals within the mitochondrial matrix. Consistently, myricetin decreased protein carbonylation in aged* sod2*Δ cells, although it had a modest effect in parental cells (Figures 4(c)–4(e)). In contrast, pyrogallol and phloroglucinol did not extend the CLS of* sod2*Δ cells (Figure 4(b)).

**Figure 5:**
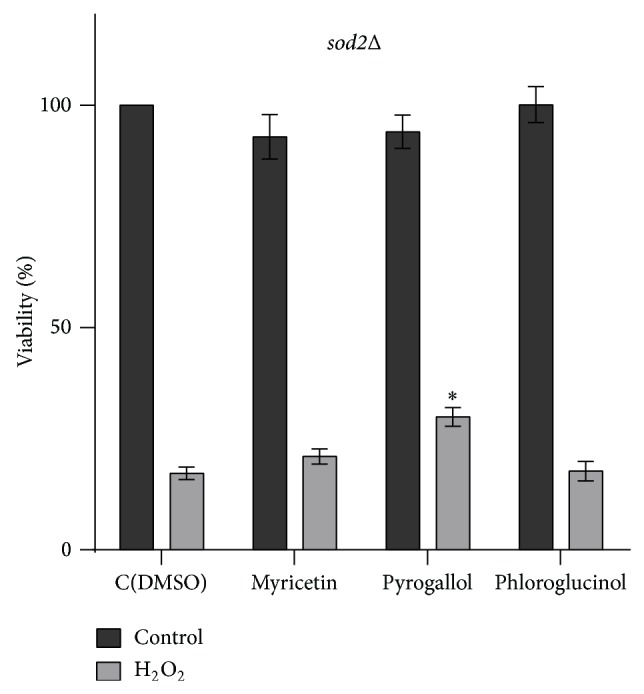
Effect of myricetin, pyrogallol, and phloroglucinol on the oxidative stress resistance of* sod2*Δ cells. Yeast cells were grown to the exponential phase in YPD medium, pretreated with compounds (300 *μ*M) or equal volume of DMSO (control) for 15 min, and subsequently treated with 1.5 mM H_2_O_2_ for 1 h. Viability is expressed as the percentage of the CFU. Values are mean ± SEM of at least 3 independent assays. Values were compared by one-way ANOVA, Dunnett's multiple comparisons test (^∗^ *p* < 0.05).

Mitochondria play an important function during oxidative stress. Indeed, *ρ*0 petite strains, which lack mitochondrial DNA, and cells deficient in electron transport chain function are sensitive to H_2_O_2_ [32, 33]. A recent study showed that H_2_O_2_ increases the mitochondrial production of superoxide radicals, which have a protective effect at low concentrations [34]. However, high concentrations of superoxide radicals are detrimental. In agreement,* sod2*Δ cells were sensitive to H_2_O_2_ (Figure 5). We also assessed the effect of polyphenols in* sod2*Δ cells exposed to H_2_O_2_. The results show that pyrogallol pretreatment slightly increased H_2_O_2_ resistance of* sod2*Δ cells, although to levels below those observed in parental cells. In contrast, myricetin and phloroglucinol did not affect H_2_O_2_ resistance in these mutants (Figure 5).

## 4. Discussion

The increased production of ROS and RNS together with the decrease of antioxidant defenses has been implicated in the pathogenesis of numerous diseases and aging [28]. Thus, a diet containing natural compounds with antioxidant properties, such as phenolic compounds, may be beneficial to human health. The antioxidant activity of these compounds is determined by structural features, including the number and position of hydroxyl groups, polarity, solubility, and reducing potential [35, 36]. In this study, we used the yeast* Saccharomyces cerevisiae* to assess* in vivo* the antioxidant capacity of the flavonol myricetin and two simple phenols, pyrogallol and phloroglucinol. Myricetin was the most effective in increasing H_2_O_2_ resistance in yeast, whereas phloroglucinol had no protective effect. Consistently, H_2_O_2_-induced intracellular oxidation and protein carbonylation decreased in cells pretreated with myricetin and pyrogallol but not with phloroglucinol. Pyrogallol and phloroglucinol contain three hydroxyl groups in the ortho- and metaposition, respectively, of a benzene ring. The vicinal positions of hydroxyl groups in pyrogallol result in a lower bond dissociation energy of O–H, facilitating the donation of hydrogen to free radicals [37]. In accordance with that, our results show that pyrogallol, in contrast with phloroglucinol, increased the viability of yeast cells exposed to H_2_O_2_. Myricetin, which contains a pyrogallol structure in the B ring, provided an even higher resistance. Our results are in accordance with data demonstrating the importance of the pyrogallol structure for the bioactivity of phenolic compounds [38]. Our data is also consistent with several reports showing a protective effect of myricetin against oxidative stress in mammalian cells. For instance, myricetin decreases H_2_O_2_-induced DNA damage in Caco-2 and HepG2 cells [39] and decreases tert-butyl hydroperoxide-induced protein oxidation and lipid peroxidation in erythrocytes from T2DM patients [40].

Being redox-active compounds, phenolic compounds can also act as prooxidants and, therefore, induce stress responses leading to an increase in the levels of cellular antioxidant defenses [41, 42]. Our results indicate that this mechanism does not contribute to the protective effects of myricetin and pyrogallol in yeast, since these compounds did not increase intracellular oxidation or affect catalase and SOD activities under the conditions used in this study. We have previously observed that hydrogen peroxide resistance in yeast incubated with quercetin is also not associated with prooxidant effects or modulation of antioxidant defenses [13]. In contrast, other reports showed that catalase activity increases in yeast treated with resveratrol and catechin, enhancing cellular resistance to oxidative stress [15].

Glutathione is an important cellular small molecule responsible for the maintenance of redox homeostasis [27]. The reduced form (GSH) mediates H_2_O_2_ decomposition catalyzed by glutathione peroxidase [27] giving rise to oxidized glutathione (GSSG) which is then reduced to GSH by glutathione reductase [43]. Glutathione has also important functions in detoxification of toxic compounds [44] and in the protection of proteins from oxidation through glutathionylation [45]. Thus, glutathione oxidation is a biomarker of oxidative stress. In control (DMSO-treated) cells, exposure to H_2_O_2_ led to an increase in GSSG levels that, concomitantly with glutathione depletion, resulted in a higher GSSG/GSH_T_ ratio. In cells pretreated with myricetin, H_2_O_2_-induced glutathione oxidation and the increase in the ratio GSSG/GSH_T_ were suppressed, which is consistent with the reduction of intracellular oxidation. Pretreatment with pyrogallol, which had a lower protective effect comparing with myricetin, did not prevent glutathione oxidation. These results suggest a correlation between the protective effect of myricetin and maintenance of glutathione redox status. Treatment with both myricetin and pyrogallol per se led to a decrease in total GSH levels, which may result from the formation of GS-compound adducts mediated by glutathione-S-transferases. Indeed, these adducts have been reported for quercetin [41, 46] and glutathione-S-transferase activity may be induced by these phenolic compounds, similarly to the effects of coumarin [47].

High levels of ROS have been implicated in aging in yeast and higher eukaryotes [48, 49]. The accumulation of oxidative damage leading to neuronal death is associated with age-related diseases such as Alzheimer's and Parkinson diseases [50]. Therefore, a diet replete in phytochemicals with antioxidant activity, characteristic of a Mediterranean diet, reduces the functional decline associated with aging and age-related disorders, increasing health span [51, 52]. Several studies showed an increase of the lifespan of yeast cells incubated with phenolic compounds. Resveratrol and phloridzin, a major apple compound, increase yeast replicative lifespan by mechanisms associated with the activation of the sirtuin Sir2p [53, 54]. Moreover, quercetin and apple polyphenolic fractions increase yeast chronological lifespan [13, 55]. Here, we report that although myricetin does not affect the chronological lifespan of parental and* sod1*Δ cells, it extends the lifespan of yeast cells lacking the mitochondrial superoxide dismutase, which are known to exhibit a very short lifespan [29]. In contrast, pyrogallol did not extend the CLS of* sod2*Δ cells. These results suggest that myricetin may be more effective in protecting aged cells that have high intracellular ROS levels and oxidative damage, especially in the mitochondria. In agreement with that, several reports show protective effects of myricetin in mitochondria. For instance, the protective effect of L-ascorbic acid against paraquat, a generator of superoxide radicals, is more pronounced in* sod2*Δ than in parental cells [56]. In addition, myricetin decreased the generation of H_2_O_2_ in isolated mouse skeletal muscle mitochondria [57] and decreased the depolarization of the inner mitochondrial membrane potential in C6 glial cells exposed to oxygen-glucose deprivation [58] and it was the most efficient among other phenolic compounds in the protection of mouse brain mitochondria against toxicity induced by methyl mercury [59]. Notably, myricetin was unable to protect* sod2*Δ cells against high doses of H_2_O_2_ whereas pyrogallol slightly increased the oxidative stress resistance of these mutants. It is likely that the excessive oxidative stress in* sod2*Δ cells treated with high doses of H_2_O_2_ overwhelms the protective effects of these compounds.

## 5. Conclusion

In summary, our data show that myricetin and, to a lesser extent, pyrogallol, increased yeast resistance to H_2_O_2_. This protective effect was correlated to a reduction in intracellular oxidation and protein carbonylation and maintenance of GSSG/GSH_T_ ratio. However, changes in catalase or superoxide dismutase activities were not associated with the protective effects. Furthermore, myricetin attenuated the shortened CLS of yeast cells lacking the mitochondrial superoxide dismutase (*sod2*Δ mutants).

## Abbreviations

CLS: Chronological lifespan
DMSO: Dimethyl sulfoxide
GSH: Reduced glutathione
GSH_T_: Total glutathione
GSSG: Oxidized glutathione
H_2_DCF-DA: 2′,7′-Dichlorodihydrofluorescein diacetate
PAGE: Polyacrylamide gel electrophoresis
RNS: Reactive nitrogen species
ROS: Reactive oxygen species
SC: Synthetic complete
SOD: Superoxide dismutase.

## References

[1] Halliwell B., Gutteridge J. (2007). Cellular responses to oxidative stress: adaptation, damage, repair, senescence and death. *Free Radicals in Biology and Medicine*.

[2] Valko M., Rhodes C. J., Moncol J., Izakovic M., Mazur M. (2006). Free radicals, metals and antioxidants in oxidative stress-induced cancer. *Chemico-Biological Interactions*.

[3] Jacob R. A., Burri B. J. (1996). Oxidative damage and defense. *The American Journal of Clinical Nutrition*.

[4] del Rio D., Rodriguez-Mateos A., Spencer J. P. E., Tognolini M., Borges G., Crozier A. (2013). Dietary (poly)phenolics in human health: structures, bioavailability, and evidence of protective effects against chronic diseases. *Antioxidants & Redox Signaling*.

[5] Stevenson D. E., Hurst R. D. (2007). Polyphenolic phytochemicals—just antioxidants or much more?. *Cellular and Molecular Life Sciences*.

[6] Crozier A., Jaganath I. B., Clifford M. N. (2009). Dietary phenolics: chemistry, bioavailability and effects on health. *Natural Product Reports*.

[7] Sies H. (2010). Polyphenols and health: update and perspectives. *Archives of Biochemistry and Biophysics*.

[8] Seifried H. E., Anderson D. E., Fisher E. I., Milner J. A. (2007). A review of the interaction among dietary antioxidants and reactive oxygen species. *The Journal of Nutritional Biochemistry*.

[9] Kong A.-N. T., Yu R., Chen C., Mandlekar S., Primiano T. (2000). Signal transduction events elicited by natural products: role of MAPK and caspase pathways in homeostatic response and induction of apoptosis. *Archives of Pharmacal Research*.

[10] Ramos S. (2008). Cancer chemoprevention and chemotherapy: dietary polyphenols and signalling pathways. *Molecular Nutrition & Food Research*.

[11] Son Y., Cheong Y. K., Kim N. H., Chung H. T., Kang D. G., Pae H. O. (2011). Mitogen-activated protein kinases and reactive oxygen species: how can ROS activate MAPK pathways?. *Journal of Signal Transduction*.

[12] Wu M. J., O'Doherty P. J., Fernandez H. R. (2011). An antioxidant screening assay based on oxidant-induced growth arrest in *Saccharomyces cerevisiae*. *FEMS Yeast Research*.

[13] Belinha I., Amorim M. A., Rodrigues P. (2007). Quercetin increases oxidative stress resistance and longevity in *Saccharomyces cerevisiae*. *Journal of Agricultural and Food Chemistry*.

[14] Vilaça R., Mendes V., Mendes M. V. (2012). Quercetin protects *Saccharomyces cerevisiae* against oxidative stress by inducing trehalose biosynthesis and the cell wall integrity pathway. *PLoS ONE*.

[15] Dani C., Bonatto D., Salvador M., Pereira M. D., Henriques J. A. P., Eleutherio E. (2008). Antioxidant protection of resveratrol and catechin in *Saccharomyces cerevisiae*. *Journal of Agricultural and Food Chemistry*.

[16] Jiménez A., Lisa-Santamaría P., García-Marino M., Escribano-Bailón M. T., Rivas-Gonzalo J. C., Revuelta J. L. (2010). The biological activity of the wine anthocyanins delphinidin and petunidin is mediated through Msn2 and Msn4 in *Saccharomyces cerevisiae*. *FEMS Yeast Research*.

[17] Martorell P., Forment J. V., de Llanos R. (2011). Use of *Saccharomyces cerevisiae* and *Caenorhabditis elegans* as model organisms to study the effect of cocoa polyphenols in the resistance to oxidative stress. *Journal of Agricultural and Food Chemistry*.

[18] Müller C., Lang R., Hofmann T. (2006). Quantitative precursor studies on di- and trihydroxybenzene formation during coffee roasting using ‘in bean’ model experiments and stable isotope dilution analysis. *Journal of Agricultural and Food Chemistry*.

[19] Yasuda T., Inaba A., Ohmori M., Endo T., Kubo S., Ohsawa K. (2000). Urinary metabolites of gallic acid in rats and their radical-scavenging effects on 1,1-diphenyl-2-picrylhydrazyl radical. *Journal of Natural Products*.

[20] Montero L., Herrero M., Ibáñez E., Cifuentes A. (2014). Separation and characterization of phlorotannins from brown algae *Cystoseira abies-marina* by comprehensive two-dimensional liquid chromatography. *Electrophoresis*.

[21] Hertog M. G. L., Feskens E. J. M., Hollman P. C. H., Katan M. B., Kromhout D. (1993). Dietary antioxidant flavonoids and risk of coronary heart disease: the Zutphen Elderly Study. *The Lancet*.

[22] Tietze F. (1969). Enzymic method for quantitative determination of nanogram amounts of total and oxidized glutathione: applications to mammalian blood and other tissues. *Analytical Biochemistry*.

[23] Flohe L., Otting F. (1984). Superoxide dismutase assays. *Methods in Enzymology*.

[24] Conyers S. M., Kidwell D. A. (1991). Chromogenic substrates for horseradish peroxidase. *Analytical Biochemistry*.

[25] Salo D. C., Pacifici R. E., Lin S. W., Giulivi C., Davies K. J. A. (1990). Superoxide dismutase undergoes proteolysis and fragmentation following oxidative modification and inactivation. *The Journal of Biological Chemistry*.

[26] Belazzi T., Wagner A., Wieser R. (1991). Negative regulation of transcription of the *Saccharomyces cerevisiae* catalase T (CTT1) gene by cAMP is mediated by a positive control element. *The EMBO Journal*.

[27] Meister A., Anderson M. E. (1983). Glutathione. *Annual Review of Biochemistry*.

[28] Dai D. F., Chiao Y. A., Marcinek D. J., Szeto H. H., Rabinovitch P. S. (2014). Mitochondrial oxidative stress in aging and healthspan. *Longevity & Healthspan*.

[29] Longo V. D., Gralla E. B., Valentine J. S. (1996). Superoxide dismutase activity is essential for stationary phase survival in *Saccharomyces cerevisiae*: mitochondrial production of toxic oxygen species in vivo. *Journal of Biological Chemistry*.

[30] Ziegler D. V., Wiley C. D., Velarde M. C. (2015). Mitochondrial effectors of cellular senescence: beyond the free radical theory of aging. *Aging Cell*.

[31] Demir A. B., Koc A. (2010). Assessment of chronological lifespan dependent molecular damages in yeast lacking mitochondrial antioxidant genes. *Biochemical and Biophysical Research Communications*.

[32] Grant C. M., MacIver F. H., Dawes I. W. (1997). Mitochondrial function is required for resistance to oxidative stress in the yeast *Saccharomyces cerevisiae*. *FEBS Letters*.

[33] Thorpe G. W., Fong C. S., Alic N., Higgins V. J., Dawes I. W. (2004). Cells have distinct mechanisms to maintain protection against different reactive oxygen species: oxidative-stress-response genes. *Proceedings of the National Academy of Sciences of the United States of America*.

[34] Thorpe G. W., Reodica M., Davies M. J. (2013). Superoxide radicals have a protective role during H_2_O_2_ stress. *Molecular Biology of the Cell*.

[35] Bors W., Michel C., Stettmaier K. (2001). Structure-activity relationships governing antioxidant capacities of plant polyphenols. *Methods in Enzymology*.

[36] Menezes J. C. J. M. D. S., Kamat S. P., Cavaleiro J. A. S., Gaspar A., Garrido J., Borges F. (2011). Synthesis and antioxidant activity of long chain alkyl hydroxycinnamates. *European Journal of Medicinal Chemistry*.

[37] Thavasi V., Leong L. P., Bettens R. P. A. (2006). Investigation of the influence of hydroxy groups on the radical scavenging ability of polyphenols. *The Journal of Physical Chemistry A*.

[38] Mitsuhashi S., Saito A., Nakajima N., Shima H., Ubukata M. (2008). Pyrogallol structure in polyphenols is involved in apoptosis-induction on HEK293T and K562 cells. *Molecules*.

[39] Aherne S. A., O'Brien N. M. (1999). Protection by the flavonoids myricetin, quercetin, and rutin against hydrogen peroxide-induced DNA damage in Caco-2 and Hep G2 cells. *Nutrition and Cancer*.

[40] Pandey K. B., Mishra N., Rizvi S. I. (2009). Myricetin may provide protection against oxidative stress in type 2 diabetic erythrocytes. *Zeitschrift für Naturforschung Teil C: Biochemie, Biophysik, Biologie, Virologie*.

[41] Kessler M., Ubeaud G., Jung L. (2003). Anti- and pro-oxidant activity of rutin and quercetin derivatives. *Journal of Pharmacy and Pharmacology*.

[42] Calabrese V., Cornelius C., Dinkova-Kostova A. T. (2012). Cellular stress responses, hormetic phytochemicals and vitagenes in aging and longevity. *Biochimica et Biophysica Acta—Molecular Basis of Disease*.

[43] Mullineaux P., Creissen G., Broadbent P., Reynolds H., Kular B., Wellburn A. (1994). Elucidation of the role of glutathione reductase using transgenic plants. *Biochemical Society Transactions*.

[44] St-Pierre M. V., Ruetz S., Epstein L. F., Gros P., Arias I. M. (1994). ATP-dependent transport of organic anions in secretory vesicles of *Saccharomyces cerevisiae*. *Proceedings of the National Academy of Sciences of the United States of America*.

[45] Gallogly M. M., Mieyal J. J. (2007). Mechanisms of reversible protein glutathionylation in redox signaling and oxidative stress. *Current Opinion in Pharmacology*.

[46] Spencer J. P. E., Kuhnle G. G. C., Williams R. J., Rice-Evans C. (2003). Intracellular metabolism and bioactivity of quercetin and its in vivo metabolites. *Biochemical Journal*.

[47] Higgins L. G., Hayes J. D. (2011). Mechanisms of induction of cytosolic and microsomal glutathione transferase (GST) genes by xenobiotics and pro-inflammatory agents. *Drug Metabolism Reviews*.

[48] Bitterman K. J., Medvedik O., Sinclair D. A. (2003). Longevity regulation in *Saccharomyces cerevisiae*: linking metabolism, genome stability, and heterochromatin. *Microbiology and Molecular Biology Reviews*.

[49] Fabrizio P., Battistella L., Vardavas R. (2004). Superoxide is a mediator of an altruistic aging program in *Saccharomyces cerevisiae*. *The Journal of Cell Biology*.

[50] Ho Y.-S., Poon D. C.-H., Chan T.-F., Chang R. C.-C. (2012). From small to big molecules: how do we prevent and delay the progression of age-related neurodegeneration?. *Current Pharmaceutical Design*.

[51] Alcalay R. N., Gu Y., Mejia-Santana H., Cote L., Marder K. S., Scarmeas N. (2012). The association between Mediterranean diet adherence and Parkinson's disease. *Movement Disorders*.

[52] Vassallo N., Scerri C. (2013). Mediterranean diet and dementia of the Alzheimer type. *Current Aging Science*.

[53] Howitz K. T., Bitterman K. J., Cohen H. Y. (2003). Small molecule activators of sirtuins extend *Saccharomyces cerevisiae* lifespan. *Nature*.

[54] Xiang L., Sun K., Lu J. (2011). Anti-aging effects of phloridzin, an apple polyphenol, on yeast via the SOD and Sir2 genes. *Bioscience, Biotechnology and Biochemistry*.

[55] Palermo V., Mattivi F., Silvestri R., la Regina G., Falcone C., Mazzoni C. (2012). Apple can act as anti-aging on yeast cells. *Oxidative Medicine and Cellular Longevity*.

[56] Saffi J., Sonego L., Varela Q. D., Salvador M. (2006). Antioxidant activity of L-ascorbic acid in wild-type and superoxide dismutase deficient strains of *Saccharomyces cerevisiae*. *Redox Report*.

[57] Grünz G., Haas K., Soukup S. (2012). Structural features and bioavailability of four flavonoids and their implications for lifespan-extending and antioxidant actions in *C. elegans*. *Mechanisms of Ageing and Development*.

[58] Panickar K. S., Anderson R. A. (2011). Mechanisms underlying the protective effects of myricetin and quercetin following oxygen-glucose deprivation-induced cell swelling and the reduction in glutamate uptake in glial cells. *Neuroscience*.

[59] Franco J. L., Posser T., Missau F. (2010). Structure-activity relationship of flavonoids derived from medicinal plants in preventing methylmercury-induced mitochondrial dysfunction. *Environmental Toxicology and Pharmacology*.

